# Supplementary material for: Implications of Heterogeneity of Epithelial-Mesenchymal States in Acromegaly Therapeutic Pharmacologic Response
Source: Biomedicines. 2022 Feb 16;10(2):460. doi: 10.3390/biomedicines10020460 (PMC8962441; doi:10.3390/biomedicines10020460)
Supplement: Supplementary file 1 [file biomedicines-10-00460-s001.zip › biomedicines-1567069-supplementary.pdf]

Supplementary Table S1. Association between the relative expression of each molecular marker and different tumor characteristics

Gene	Relative expression mean ± SE		p-value
**TUMOR SIZE**			
	**< 2cm Ø** *n = 34*	**> 2cm Ø** *n = 21*	
SNAI1	0.038 ± 0.007	0.044 ± 0.015	0.780
SNAI2	0.110 ± 0.030	0.266 ± 0.173	0.431
ESRP1	1.119 ± 0.128	0.748 ± 0.241	0.267
VIM	3.862 ± 2.148	4.399 ± 2.251	0.924
TWIST	0.015 ± 0.004	0.016 ± 0.009	0.951
	Pretreated patients		
	n = 29	*n = 16*	
RORC	1.256 ± 0.157	1.197 ± 0.229	0.834
N-cadherin	0.425 ± 0.128	0.154 ± 0.061	**0.035**
	Non-pretreated patients		
	n = 5	*n = 5*	
RORC	0.672 ± 0.147	0.316 ± 0.077	0.247
N-cadherin	0.425 ± 0.128	0.140 ± 0.054	**0.047**
**EXTRASELLAR GROWTH**			
	**NO** *n = 18*	**YES** *n = 39*	
SNAI1	0.025 ± 0.005	0.047 ± 0.010	**0.049**
SNAI2	0.116 ± 0.039	0.195 ± 0.095	0.444
ESRP1	1.089 ± 0.198	0.923 ± 0.148	0.507
VIM	5.640 ± 4.173	3.424 ± 1.252	0.617
TWIST	0.011 ± 0.004	0.017 ± 0.005	0.418
	Pretreated patients		
	n = 16	*n = 32*	
RORC	1.252 ± 0.205	1.227 ± 0.160	0.914
N-cadherin	0.413 ± 0.197	0.281 ± 0.082	0.554
	Non-pretreated patients		
	n = 3	*n = 7*	
RORC	0.445 ± 0.189	0.558 ± 0.203	0.697
N-cadherin	0.100 ± 0.059	0.130 ± 0.054	0.716
**CAVERNOUS SINUS INVASION**			
	**NO** *n = 28*	**YES** *n = 26*	
SNAI1	0.035 ± 0.008	0.046 ± 0.012	0.500
SNAI2	0.131 ± 0.037	0.211 ± 0.140	0.582
ESRP1	1.050 ± 0.161	0.770 ± 0.167	0.232
VIM	4.496 ± 2.571	3.847 ± 1.871	0.839
TWIST	0.012 ± 0.004	0.020 ± 0.008	0.399

	Pretreated patients		
	n = 21	*n = 23*	
RORC	1.375 ± 0.206	1.068 ± 0.187	0.234
N-cadherin	0.333 ± 0.146	0.337 ± 0.101	0.982
	Non-pretreated patients		
	n = 7	*n = 3*	
RORC	0.482 ± 0.144	0.046 ± 0.026	**0.028**
N-cadherin	0.129 ± 0.055	0.131 ± 0.129	0.991
	***GNAS* MUTATION**		
	**NO**	**YES**	
	n = 18	*n = 13*	
SNAI1	0.052 ± 0.012	0.049 ± 0.023	0.923
SNAI2	0.223 ± 0.055	0.336 ± 0.279	0.698
ESRP1	1.068 ± 0.228	1.119 ± 0.260	0.884
VIM	7.436 ± 4.356	4.881 ± 3.619	0.656
TWIST	0.022 ± 0.006	0.024 ± 0.015	0.885
	Pretreated patients		
	n = 14	*n = 8*	
RORC	1.081 ± 0.185	1.331 ± 0.225	0.217
N-cadherin	0.808 ± 0.245	0.047 ± 0.023	**0.007**
	Non-pretreated patients		
	n = 4	*n = 5*	
RORC	0.576 ± 0.199	0.596 ± 0.256	0.954
N-cadherin	0.004 ± 0.001	0.155 ± 0.058	0.058
	**HYPOINTENSE T2 SIGNAL**		
	**NO**	**YES**	
	n = 35	*n = 17*	
SNAI1	0.039 ± 0.007	0.044 ± 0.018	0.822
SNAI2	0.109 ± 0.030	0.291 ± 0.213	0.409
ESRP1	0.864 ± 0.132	1.047 ± 0.242	0.511
VIM	2.063 ± 0.365	8.139 ± 4.643	0.210
TWIST	0.119 ± 0.003	0.222 ± 0.011	0.395
	Pretreated patients		
	n = 29	*n = 15*	
RORC	1.365 ± 0.174	1.072 ± 0.163	0.226
N-cadherin	0.219 ± 0.054	0.402 ± 0.200	0.390
	Non-pretreated patients		
	n = 6	*n = 2*	
RORC	0.482 ± 0.144	0.046 ± 0.026	**0.028**
N-cadherin	0.129 ± 0.055	0.131 ± 0.129	0.991

P-value was calculated for each tumor characteristics (Student's t test or Mann–Whitney U test, as appropriate). Significant p-values are shown in bold. In the case of N-cadherin and *RORC,* whose expression is affected by presurgical SRLs treatment, the analysis was performed segregating pretreated and non-pretreated patients. Abbreviations: SE, Standard Error.
